# Problems with Using the Normal Distribution – and Ways to Improve Quality and Efficiency of Data Analysis

**DOI:** 10.1371/journal.pone.0021403

**Published:** 2011-07-14

**Authors:** Eckhard Limpert, Werner A. Stahel

**Affiliations:** 1 ELI-o-Research, Life Sciences, Zurich, Switzerland; 2 Seminar for Statistics, Swiss Federal Institute of Technology (ETH) Zurich, Zurich, Switzerland; University of Glasgow, United Kingdom

## Abstract

**Background:**

The Gaussian or normal distribution is the most established model to characterize quantitative variation of original data. Accordingly, data are summarized using the arithmetic mean and the standard deviation, by 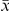 ± SD, or with the standard error of the mean, 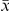 ± SEM. This, together with corresponding bars in graphical displays has become the standard to characterize variation.

**Methodology/Principal Findings:**

Here we question the adequacy of this characterization, and of the model. The published literature provides numerous examples for which such descriptions appear inappropriate because, based on the “95% range check”, their distributions are obviously skewed. In these cases, the symmetric characterization is a poor description and may trigger wrong conclusions. To solve the problem, it is enlightening to regard causes of variation. Multiplicative causes are by far more important than additive ones, in general, and benefit from a multiplicative (or log-) normal approach. Fortunately, quite similar to the normal, the log-normal distribution can now be handled easily and characterized at the level of the original data with the help of both, a new sign, ***^x^***/, times-divide, and notation. Analogous to 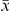 ± SD, it connects the multiplicative (or geometric) mean 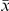 * and the multiplicative standard deviation s* in the form 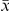 * ***^x^***/s*, that is advantageous and recommended.

**Conclusions/Significance:**

The corresponding shift from the symmetric to the asymmetric view will substantially increase both, recognition of data distributions, and interpretation quality. It will allow for savings in sample size that can be considerable. Moreover, this is in line with ethical responsibility. Adequate models will improve concepts and theories, and provide deeper insight into science and life.

## Introduction

Quantitative variation in scientific data is usually described by the arithmetic mean and the standard deviation in the form 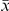 ± SD. In graphical displays, error bars around mean values display the degree of precision of the means – which is usually essential for an adequate interpretation. This characterization is adequate for and evokes the image of a symmetric distribution or, more specifically, the normal or Gaussian distribution [1]–[3]. As is well known, the latter model implies that the range from 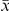 - SD to 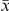 + SD contains roughly the middle two thirds (68%) of the variation, and the interval 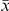 ± 2 SD covers 95%. So widely is this description used that it is almost mandatory in most scientific journals to present data with their means and either standard deviations or standard errors of the mean (SEM), in the form 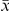 ± SD or 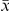 ± SEM.

## Results and Discussion

### The Problem

However, there are numerous examples for which the description by a mean and a symmetric range of variation around it is clearly misleading. This becomes obvious whenever the standard deviation is of the same order as the mean so that the lower end of the 95% data interval extends below zero for data that cannot be negative, as is the case for most original data in science. In such cases, we say that the data fail the “95% range check.” Table 1a presents some recent examples. For instance, in investigations of health risk, a sample of insulin concentrations in rat blood is described by 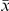 ± SD  =  296±172 (4]. If a normal distribution were appropriate, the 95% range would extend from -48 to 640, and 4% of the animals would have negative insulin values which is, of course, impossible. Moreover and worse, in this and many further examples, there is even a positive threshold below which values cannot occur. Clearly, data of this kind will be skewed.

**Table 1:** Misleading characterization of data.

Discipline					
Character	Case	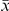 ± SEM (n)	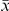 ± SD	95% ( 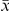 ±2 SD)	Reference
a) Cases based on SD					
**Medicine**					
Risk factors	A- Insulin, pM		*296±172*	**-48** to 640	[4] Table 1
	B- Running capacity, m		*700±400°*	**-100** to 1′500	[4] Fig. 1, HCR, Gen.8
**Biology**					
Genetics	C- KAP1, Mest, % tot. input		*2.0±1.9°*	**-1.75** to 5.85	[7] Fig 3b
Cytology	D- Exon expres., leukocytes		*15.2±12.7°*	**-10.2** to 40.5	[8] Fig 4 A
Phytopathology	E- Fungic. sensitivity, mg l^−1^		*25.0±* ***26.4***	**-27.8** to 77.8	[9], Tab. 2
b) Cases based on SEM					
	F- Cells/ml, x 10^6^	*0.25±0.16 °* (3)	0.25±**0.28**	**-0.30** to 0.80	[5] Fig. 7, E2
Tumorigenesis	G- Microadenomas	*2.06±1.63* (4)	2.06±**3.26**	**-4.46** to 8.58	[10] p125, line –18
**Marine ecology**					
	H- Cell density	*6000±4400°* (3)	6000±**7621**	**-9242** to 21242	[11] Fig 4 (6 days)
**Soil Science**					
Deforestation	I- Calc. P, (kg/ha)	*62±48*° (3)	62±**83**	**-104** to 228	[12] Fig 1B, 0 cycles
**Food Science**					
Honey	J- HMF-content, mg/kg	10.1±0.3 (1573)	10.1±**11.8**	**-13.5** to 23.7	after [13]

**a,** Frequently, variation in data from across the sciences is characterized with the arithmetic mean 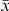 and the standard deviation SD. Often, it is evident from the numbers that the data have to be skewed. This becomes clear if the lower end of the 95% interval of normal variation, 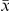 - 2 SD, extends below zero, thus failing the “95% range check”, as is the case for all cited examples. Values in **bold** contradict the positive nature of the data. **b**, More often, variation is described with the standard error of the mean, SEM (SD  =  SEM · √*n*, with *n*  =  sample size). Such distributions are often even more skewed, and their original characterization as being symmetric is even more misleading. Original values are given in italics (°estimated from graphs). Most often, each reference cited contains several examples, in addition to the case(s) considered here. Table 2 collects further examples.

**Table 2:** Summarizing data – Problems and solutions.

Discipline		Description, original		95% range	Description, recommended^1^		95% range
Subject	Case, reference	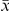 ± SEM (n)	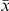 ± SD	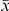 ± 2 SD	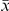 * ^x^/SEM*	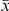 * ^x^/s*	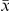 * ^x^/(s*)^2^
**Medicine**	Concentration of insulin, pM, Table 1 in [4]	-	*296±172*	**-48** to 640	-	256 ^x^/1.71	87 to 753
Health risk	Running capacity, m, Fig. 1, HCR, gen. 8 in [4]	-	*700±400°*	**-100** to 1′500	-	608 x/1.7	210 to 1760
	Insulin, 30 min, SD, Fig. 2C in [23]	*1.5±0.6° (5)*	1.5±1.34	**-1.18** to 4.18	1.12 ^x^/1.41	1.12 ^x^/2.15	0.242 to 5.18
	nflammation, histological score, Fig 3F, GP6 in [42]	*1.69±0.40° (25)*	1.69±**2.00**	**-2.31** to 5.7	1.091 ^x^/1.21	1.091 ^x^/2.55	0.17 to 7.1
	Inflammation in mice, mRNA expr., Fig 7b in [43]	*6.3±3.85° (3)*	*6.3±6.7*	**-7.0** to 19.6	4.33 ^x^/1.65	4.33 ^x^/2.4	0.76 to 24.5
Tryptophan-catabolism	Kynurenine µM, Fig 2d, [44]	-	*0.4±0.3°*	**-0.2** to 1.0	-	0.32 ^x^/1.95	0.0841 to 1.22
Immune response	TNFα mRNA production, Fig. 4F, 0h, [45]	-	*0.45±0.45°*	**-0.45** to 1.35	-	0.318 ^x^/2.3	0.0602 to 1.68
Tumorigenesis	Microadenomas, frequency, p 125, line –18, [10]	*2.06±1.63° (4)*	2.06±**3.26*°***	**-4.46** to 8.58	1.1 ^x^/1.75	1.1 ^x^/3.06	0.117 to 10.3
	PCNA-positive cells, %, WT, 4 weeks, Fig 2A, [46]	*4.2±1.7 (5)*	4.2*±3.8*	**-3.4** to 11.8	3.11 ^x^/1.41	3.11 ^x^/2.17	0.66 to 14.6
**Biology**	KAP1, Mest, % total input, Fig 3b, [7]	-	*2.0±1.9°*	**-1.8** to 5.8	-	1.45^ x^/2.23	0.29 to 7.2
Genetics	D- Exon expres., leukocytes, Fig 4A, above, [8]	-	*15.2±12.7°*	**-10.2** to 40.5	-	11.64 ^x^/2.07	2.72 to 49.8
Cytology	Fus3ch concentration, nM, Fig. 2, [47]	-	*197±190°*	**-183** to 577		142 ^x^/2.25	28 to 718
	Number of cells/ml x 10^6^, Fig. 7, E2, [5]	*0.25±0.16° (3)*	0.25±**0.28**	**-0.30** to 0.80	0.167 ^x^/1.68	0.167 ^x^/2.45	0.028 to 1.0
Evolution	Living rotifers, no., after 3d, Fig. 2A, wind disp., [48]	*30±19° (17)*	30±**78.3**	**-127** to 187	10.7 ^x^/1.42	10.7 ^x^/4.2	0.6 to 189
Virology	Virus release, x10^3^, Fig. 2C, Cep55, [49]	-	*40±25°*	**-10** to 90	-	33.9 ^x^/1.78	10.8 to 107
Neurology	Labled gran. Cells, %, Fig 2G, iiC, [50]	-	*4±2.5°*	**-1** to 9	-	3.39 ^x^/1.78	1.1 to 10.7
	Freezing kinet., %, Fig 4B, 30s, *f*NR1, [51]	*15±5° (12)*	15±**17**	**-19** to 49	9.92 ^x^/1.3	9.92 ^x^/2.48	1.61 to 61.1
	*Drosophila,* Lunge numbers, Fig. 2d, 2^nd^ col., [52]	*38±17° (19)*	38±**74**	**-110** to 186	17^ x^/1.33	17^ x^/3.5	1.4 to 212
Parasitology	Luciferase +activity, x 10^6^, Fig 4e, [53]	-	*210±190°*	**-170** to 590	-	156 ^x^/2.17	33.2 to 731
Ontogeny	Cell surv. with gremlin, Fig 3C, CFU-M, [54]	-	*22±12°*	**-2** to 46	-	19.3 ^x^/1.67	6.96 to 53.6
Photosynthesis	Nitrite cons., mM, Fig 1, after 8d, [55]	-	*0.2±**0.3°***	**-0.4** to 0.8	-	0.111 ^x^/2.96	0.013 to 0.973
Signal transduction	Fluorescence, Fig 1C, untreated, 4h, [56]	*3±6° (10-20: 14)*	3±22	**-41** to 47	0.405 ^x^/1.71	0.405 ^x^/7.4	0.0074 to 22.2
Fertility, in mice	Ovulated oocytes/CD9^+/+^mice, Tab.1, [57])	*29.6±15.3 (42)*	*29.6±**99.2***	**-169** to 228	8.46 ^x^/1.28	8.46 ^x^/4.87	0.357 to 200
in plants	Transcript quantity, Fig 2C, [58]	*2.5±1.5° (3)*	2.5±**2.6**	**-2.7** to 7.7	1.73 ^x^/1.64	1.73 ^x^/2.35	0.313 to 9.6
Quiescense	Latency, s, p 571-left, line 23, [20]	*7±2 (15)*	7±**8**	**-9** to 23	4.61 ^x^/1.27	4.61 ^x^/2.49	0.741 to 28.7
**Phytopathology**	Bacteria in rhizosphere, 15d x 10^3^, [59]	*55±13 (10)*	55±41.1	**-27.2** to 137.2	44.1 ^x^/1.23	44.1 ^x^/1.95	11.6 to 167
Cell counts	*Ps. savastanoi*, CFU x 10^6^, Tab. 2, Bagno, [60]	*61±59 (8)*	61±**170**	**-279** to 401	20.6 ^x^/1.68	20.6 ^x^/4.36	1.08 to 392
Fungicide sensitivity	Botrytis cinerea – triadimenol, µg ml^-1^, p 173, [61]	*-*	*4.1±3.7*	**-3.3** to 11.5	-	3.04 ^x^/2.16	0.65 to 14.3
	Wheat p. mildew – fenpropimorph, mg l^-1^, [9]		*25±**26.4***	**-27.8** to 77.8	17.2 ^x^/1.09	17.2 ^x^/2.38	3.04 to 97.1
**Aerobiology**	Colony forming units per m^3^ air x 10^6^, [62]	*-*	*582±510*	**-582** to 1602	-	438 ^x^/2.13	96.7 to 1981
	*H. annosum*-caused gaps in forests, m^2^, [63]	*-*	*2898±1898*	**-898** to 6794	-	2424 ^x^/1.82	734 to 8008
**Marine ecology**	Data indicated at log-scale, Fig. 4, 2. col., [11]	*6000±4400° (3)*	6000±**7621**	**-9242** to 21242	3712 ^x^/1.76	3712 ^x^/2.66	523 to 26355
Nitrate in foraminifers	Boliv. subaen., Bay of B., pmol per cell, Tab. 1, [64]	*285±46 (47)*	285±**315**	**-346** to 916	191 ^x^/1.14	191 ^x^/2.45	32 to 1143
**Soil Science**	Deforestat. Calc. Pi, kg/ha, Fig. 1B, 0 cycles, [12]	*62±48° (3)*	62±**83**	**-104** to 228	37.1 ^x^/1.80	37.1 ^x^/2.75	4.89 to 282
**Physics**	Reynolds stress, β, x 10^-6^, Fig.4, bottom right, [65]	*-*	*0.5±**3.5°***	**-6.5** to 7.5	-	0.0707 ^x^/7.23	0.0014 to 3.69
**Food Sciences**	HMF-content in honey, mg/kg, after [13]	10.1±0.3 (1573)	10.1±**11.8**	**-13.5** to 23.7	*6.57* ^x^/1.02	*6.57* ^x^/2.53	1.03 to 42
^1^ These results were calculated, starting from 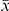 ± SD, by 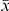 /√ω and exp(√log(ω)), respectively, where ω = 1+(SD/ 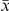 )^2^ (5). The multiplicative standard error is SEM* = (s*)^1/√^							

The collection of datasets in Table 1 is extended, and their more meaningful and, thus, recommended, descriptions based on multiplicative means and multiplicative standard errors or standard deviations are given. Some comparisons appear to be of interest. Necessarily, arithmetic means exceed multiplicative ones, starting from some 15% for small s*s around 1.7 up to more than the sevenfold for s* >7. The lower limits of the 95% ranges, relative to the means, turn increasingly negative with s* growing for the classical version, but remain positive and get smaller for the multiplicative description. Turning to upper limits, the multiplicative limit exceeds the additive one by some 17% for s*  =  1.7. With s*  =  2.5, the difference is about 25%. For s*  =  4.2, there is no difference, and for s*  =  7, the additive mean is only half the multiplicative one.

The problem is less apparent, but often even more severe if, instead of standard deviations, standard errors of the mean (SEM) are given (Table 1b). In such cases the intervals obtained, compared to the mean value, are shorter, thus hiding the skewed nature of the data.

One example is on data evaluation and error bars and gives helpful explanations of several points of confusion on this topic [5]. It is highly estimated and one of the *top ten of all-time most viewed papers in biology* according to the Faculty of 1000 [6], In this paper, symmetric error bars showing SEM of *n*  =  3 observations are displayed for data sets concerning the evolution of clonal cell counts, but for 3 out of 8 samples (E2-E4), the estimated distribution, if assumed normal, would suggest between 12% and 19% of the data being negative.

A peculiar type of plot is found in [10] (Fig. 4, p. 471). Based on the established symmetric view at the level of the original data as described above, the means and standard errors (of *n*  =  3) are presented in this case on a logarithmically scaled vertical axis. This results in asymmetric intervals with upward bars that are shorter than downward ones. Again, as a 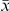 ± 2 SD interval would enclose negative numbers in at least one case, the corresponding lower bar would extend to minus infinity on that plot.

Initially, we noticed such examples from the fields of our own research [9], [14], [15]. Extending the scope, we recognized them to exist across the sciences, with the notable exception of some fields of research such as atmospheric, hydrological, soil, or financial sciences. As a general rule, we found one or more papers with such examples per issue of a journal, including the most prestigious ones with their spectrum of contributions from across the sciences and their qualified refereeing systems. A conservative estimate based on the Journal Citation Report [16] thus leads to more than one thousand such papers published per week in the Science Edition only.

The description of data by 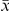 ± SD or 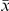 ± SEM does, of course, not formally imply the assumption of a symmetrical distribution, and many authors will be aware of the asymmetric nature of their data. Then, for any formal analyses of the data, appropriate methods, notably nonparametric tests, are used. In the same paper, however, graphical displays usually still use the symmetric description, thus pointing to a dilemma. In any case, our emphasis here is not to criticize inadequate analyses of data, but to highlight the potential for improved quality and new insights to be obtained by using an alternative description.

### Towards Solving the Problem

**Figure 1:**
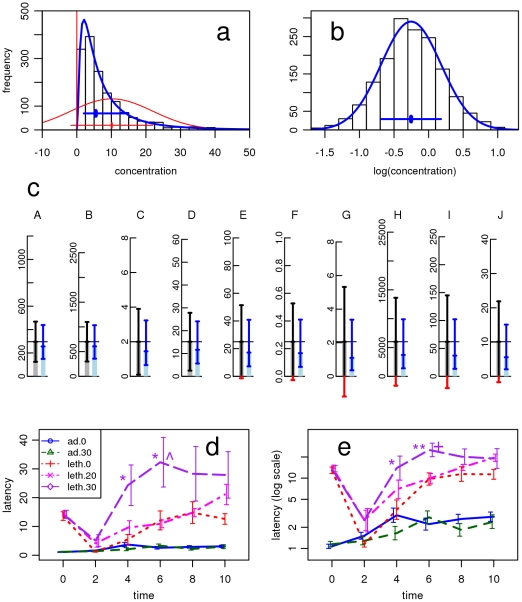
Adequate characterization of data improves the results. - **a,b**, The frequency distribution of a chemical (hydroxymethylfurfurol, HMF) in honey is used to illustrate the problem and its solution. **a.** Obviously, the normal density curve does not fit this skewed dataset, but the log-normal does. **b.** the distribution is normal after logarithmic transformation and, thus, log-normal. Back-transforming 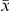 and SD from the level of the logarithms gives the multiplicative (or geometric) mean 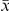 * and the multiplicative standard deviation s* that allow to characterize variation at the original scale of the data **(a)**, **c**, Comparing the two types of (1 standard deviation) intervals for the datasets A-J shown in Table 1. Clearly, the multiplicative intervals are shorter, increasing, thus, the potential for differentiation. Moreover, they never lead to negative values, and usually describe the variation encountered well. **d,e**, Multiplicative intervals improve differentiation in an example from [20]. **d**, Original, additive description of variation, with two significant differences, *, and a third one, close to significance. Error bars indicate SEM. **e**, The multiplicative type of intervals (based on the original, unpublished data received from the authors) shown here with a log-scale on the vertical axis leads to a more plausible picture, makes all three differences more significant, and one highly significant now. Error bars indicate SEM*.

In all cases cited in Table 1, the distributions of the datasets will be skewed, with the longer tail to the right. The simplest model that describes such variability is the log-normal distribution [12], [17]–[19]. Fig. 1a shows a typical case of data (last line in Table 1) with fitted normal and log-normal distributions. The normal distribution is clearly inappropriate as it suggests a probability of 20% for negative values. The log-normal model corresponds to a normal distribution for logarithmically transformed data, which yields a nice fit (Fig. 1b).

Log-normal variation is most adequately characterized by the geometric - or *multiplicative* - mean 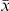 * and the multiplicative standard deviation s* [18]. These parameters determine an interval containing 2/3 of the data as does the description 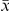 ± SD for (*additive*) normal data: The interval ranges from 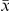 * divided by s* to 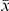 * times s* and may be denoted by 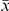 * **^x^**/s* (read “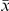 * times – divide s* ”). The two types of intervals are indicated in Fig. 1a. They are compared for all datasets of Table 1 in Fig. 1c. (Since we do not have access to the original data, 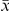 * and s* were calculated from 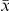 and SD using the formulas for the expectation and standard deviation of a log-normal distribution, as described in the footnote of Table 2.) The 95% variation interval for insulin in rats [4] now covers the range 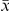 * ^x^/(s*)^2^  =  256 ^x^/(1.71)^2^  =  87 to 753 pM, that appears physiologically plausible. For the respective values and intervals for the other cases, see Table 2, which contains examples from a variety of fields of science.

[Table 2 about here.]

In order to show the advantages of an appropriate description, we discuss a graph of Raizen et al. [20] reproduced in Fig. 1d. The symmetrical error bars follow the typical pattern of skewed distributions discussed above. Using a log scaled vertical axis (Fig. 1e), the variation in the lower curves appears similar to the scatter in the upper part, thus reflecting a common relative variation for all the conditions and groups. This insight leads to more efficient statistical testing. The three two group t-tests indicated by the authors (Fig. 1d) become more significant as the p values decrease from 1.8% to 1.5%, from 4.2% to 0.5%, and from 5.3% to 2.0%, if the t-test on the original data is replaced by the same test on log transformed data (Fig. 1e). Thus, in this example, which stands for many analyses found in science, recognition of the log-normal nature of the data leads to more informative graphs and more precise statistics.

### The Fundamental Role of Multiplication - and of the Log-Normal Distribution

Heath [21] pointed out that for “certain types of data the assumption that the data are drawn from a normal population is usually wrong, and that the alternative assumption of a log-normal distribution is better”. As further explained below, this statement appears to be of a much broader importance: it is in line with the fact that, in general, laws and processes in science and life are rather of multiplicative than additive nature. From the ample evidence (e.g. 22], let us mention some basic features:

Chemistry is fundamental for life. The velocity of the reaction of A with B is proportional to the *product* of the individual concentrations, like *v* ∼ [A] • [B]. With the complex networks of biochemical reactions and pathways for, *e.g*., anabolism, catabolism, and signalling within the many kinds of biological tissues, this type of law thus affects innumerable aspects of life such as, e.g., concentrations of insulin [4], [23]. Secondly, life depends on processes and laws of mobility and permeability. Baur [24] demonstrated with thorough documentation these processes not to fit the normal, but the log-normal distribution. – Similarly, the Hagen-Poiseuille law *V_t_  = * (*ΔP Τ^4^ π)/(8 η L)* is important for mobility and, without going into detail here, consists of several *multiplicative* (and divisive) steps.

Thirdly, considering growth, it appears that *rates* are often constant in first approximation, meaning that the current size is *multiplied* by the rate to obtain the new size. Finally, cell numbers after division follow the exponential row 1-2-4-8-16. With a median concentration of, *e.g.*, 10^6^ bacteria, one cell division more or less yields 2×10^6^ or 0.5×10^6^ bacteria. The variation is asymmetric and could be described by 10^6^ ^x^/2. This appears to be the reason why for blood cell counts Sorrentino arrived at a log-normal fit [25]–[27] which is supposed to hold for other cell counts, too [e.g. 5,11,52,54,57,59,60]. In the present context, the name of one outcome of cell division is interesting to consider, as that process is simply called *multiplication.* – Summarizing, more than 50% of the examples from Table 2 can be based on one or the other of these effects, and for other examples, further multiplicative effects are quite plausible.

The link between multiplicative processes and the log-normal distribution is straightforward: Whereas additive effects lead to the normal distribution according to the Central Limit Theorem (CLT) in its additive form, that is well known and almost exclusively considered so far, the superposition of many small random *multiplicative* effects results in a log-normally distributed random variable according to the *multiplicative CLT* [17] that needs to become better known, and understood. To this aim, statistical models resembling gambling machines can help. Whereas the mechanical equivalent of the additive CLT is the established Galton board [28], the multiplicative CLT can be visualized by an analogous novel board [18], [29], [30].To conclude, there is a sound theoretical justification for thinking in multiplicative terms and using the log-normal distribution as first choice, at least as an approximation.

In addition to Heath, Baur and Sorrentino [21], [24]–[27] several authors have stressed the need for the log-normal view in their fields of research. Kelly [31], described them for food webs, and Hattis *et al*. [32] related health risks caused by toxicants to a chain of multiplicative steps including contact rate, uptake as a fraction of contacts, general systemic availability etc. Morrison [33], re-analysing published data based on using the normal view, even came, with the log-normal view, to conclusions contradicting the original ones.

There is also a more general area of concern. It relates to technical norms and limits of intervention. One example comes from testing construction material, where procedures to date are based on a normal approach, but Schäper shows the log-normal to fit better [34]. Similar considerations relate to limits of medical and chemical intervention [32], areas that appears to be of considerable concern.

In some sense, the skewed distributions failing the “95% range check” form the visible tip of the iceberg, which itself consists of the predominant multiplicative effects. A question even arises about the relevance of additive effects – and therefore of the normal distribution – in nature and science at large.

### Normally Distributed Data

Of course, there are sets of original data that can be adequately described by a normal distribution. Such samples generally have a low coefficient of variation, and the fitted log-normal and normal distributions are similar. However, since the log-normal fits many skewed samples in addition, it is to be preferred because it describes more often data adequately than the common normal distribution. Re-examining published original data, we did not find any samples fitting the (*additive*) normal distribution that did not fit the log- or *multiplicative* normal distribution equally well, or better. This even applies to examples such as body heights used in textbooks to illustrate the normal distribution. RA Fisher's data of 1164 men [1] yield a p value of a Chisquare goodness of fit of 0.13 for the normal, and of 0.48 for the log-normal distribution. Exceptions to these findings are measurements that can adopt negative values, like angles and geographical coordinates. In addition, of course, transformed data and other quantities derived from original data often show a normal distribution.

It is common practice to first perform a goodness of fit test for normality of the data and to transform the data or use an alternative to the t test if the normal distribution is rejected. Note that this recipe is not supported by statistical theory, one reason being that for small samples, the goodness of fit tests have low power to detect any deviations and will therefore rarely lead to the appropriate test. Nevertheless, we have shown above that the “95% range check” can reject normality even for very few observations.

### Increased Efficiency

Empirical studies are not only conducted to describe the data, but also to draw formal inference. The simplest and most common statistical problem is the comparison of two groups of data. To this aim, graphical descriptions are often augmented by asterisks indicating statistically significant differences. The description by 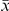 ± SD or 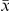 ± SEM suggests the application of the t-test as the natural choice. More careful authors apply the nonparametric Wilcoxon rank sum test instead if there are enough observations (>4) in each group. The appropriate alternative for small samples consists of applying the t-test to logarithmically transformed data. The widespread multiple comparisons procedures should also be used on transformed data.

**Figure 2:**
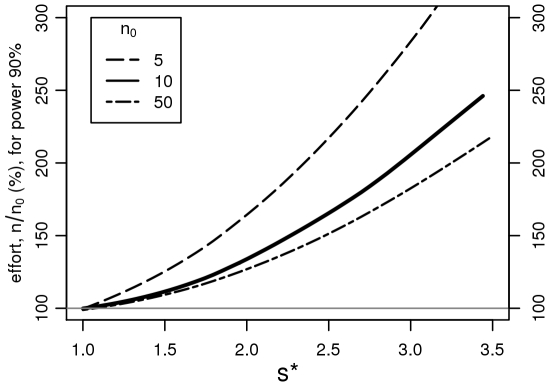
Savings in sample size. If the t-test, which is based on the normal distribution, is applied to (skewed) raw data, the statistical power is lower than for the optimal procedure, which consists of applying it to the log transformed values. Starting from 2 groups of log-normal data with a given s*, we calculate the sample size needed in each group to achieve the same (simulated) statistical power with the (inappropriate) t-test applied to the raw data as with the optimal test, applied to *n_0_*  =  5, 10, and 50 observations in each group. This sample size is a function of s*. For the median skewness, s*  =  2.4, 16 observations are needed instead of 10, corresponding to 60% additional effort.

Fortunately, the use of the t-test for skew data usually keeps the level of the test at or below the assumed level (of usually 5%). Its use entails, however, the need for more experimental data to achieve the same precision in conclusions, i.e., the power of the test is unnecessarily low. Figure 2 makes this point clear. Assuming two samples of, *e.g.*, *n_0_*  =  10 log-normal observations with a given s*, the difference of parameters 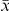 * between the two populations was chosen such that the statistical power of the adequate t-test for logarithmically transformed data is 90%. When the t-test is applied to the untransformed data, significance is obtained less often, i.e., the power is less than 90%. We therefore increased the sample size and simulated again, until the (inappropriate) test achieved the power of 90%. This increased sample size depends on the multiplicative standard deviation s*, which characterizes the skewness of the data and on the original sample size *n_0_* as shown in Fig. 2.

For our examples chosen arbitrarily (Table 2) *n* varied from 3 to 47 and was most often around 10. s* varied from 1.7 to 8.6, with 20% of the cases being above 3.1, and with a median s* of 2.4. For the latter and *n_0_*  =  10, a sample size of 16 is needed with the inappropriate way of testing to achieve the same power. This means an increase of 60% in sample size. The range of this curve, *n_0_*  =  10, starts from an increase of 20% at s*  =  1.7, and as much as 120% additional effort would be needed with s*  =  3.1. For *n_0_*  =  50 the curve is little different at the beginning and rises to 80% additional effort at s*  =  3.1. The difference in effort is most expressed with low sample size. Whereas for *n_0_*  =  5 and s*  =  1.7 there is an increase of 35%, it rises up to 200% for s*  =  3.1. Thus, for clearly skewed data, adequate evaluation leads to large savings in experimental effort, i.e., in cost, patients, or animals involved, and therefore has ethical and political relevance.

### More Precise Models

Of course, the log-normal distribution is not always the best model for skewed data. It is clearly appropriate to select a model that describes the variation of data as precisely as possible in any given application, and to use the corresponding optimal inference procedures. For some fields of science, there is solid theoretical and empirical justification to use a particular type of distribution, *e.g.*, the Weibull, Gamma, Pareto, or Exponential distribution in insurance and reliability.

Note that large samples are needed to select between different types of distributions empirically. If such data is not available, nonparametric tests and respective confidence intervals should be used. Nevertheless, in most cases the description by 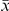 * ^x^/s* is still more adequate than 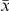 ± SD, and the log-normal model may serve as an approximation in the sense that many scientists perceive the normal as a valid approximation now.

### Conclusions and Outlook

In the light of the examples considered, it is evident that data often follow asymmetric variation, even though they are characterized in symmetric terms, and the question arises: Has the normal distribution become too normal?

We advocate the use of the log-normal distribution and the description by 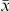 * ^x^/s* as a simple standard way of treating data — unless more adequate specific distributions are available – in the same spirit as the normal distribution and the 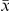 ± SD notation have been and are used up to date. In the same way, 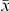 * ^x^/SEM* should replace 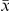 ± SEM when calculating “inferential error bars” [5], and similarly for confidence intervals.

In fact, when assessing the variability of data from the 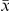 ± SD characterization, we usually compare the SD to the mean. The multiplicative standard deviation does not need such a standardisation, and there is evidence that typical values occur within most kinds of empirical data. Incubation times of human diseases, e.g., show a typical range of s* values around 1.4 [18 and Limpert & Stahel, unpublished), and it would well be of interest to see how this compares to diseases of animals and plants. Thus, the use of s* has the potential of providing deeper insight into the variability of data than the usual standard deviation.

The use of the log-normal model is equivalent to first subjecting the data to the log transformation and then proceeding with methods based on the normal distribution. In graphical displays, the use of logarithmically scaled axes combines the advantages of appropriate symmetrical error bars with the ease of interpretation of the shown values (cf. Fig 1e).

When multiplicative effects are quantified by experiments, a version of analysis of variance with multiplicative instead of additive effects would be adequate as already recognized by Fisher and Mackenzie in 1923 [35]. Such models are again akin to treating log-transformed data by usual, additive analysis of variance or regression methods. This is in agreement with the established advice of John Tukey to use logarithms as the “first aid transformation” in the evaluation of the usual type of quantitative data–-a type of data that he calls “amounts” [36]. When fitting such models, it is well known that assessing the distribution of residuals is important, and we get the impression that this point is often neglected by those who use the models for untransformed original data.

In economics and even more so in finance, the log-normal distribution has been generally used for half a century now [17], [37]–[39]. This often occurs implicitly through studying, *e.g.*, logarithmically transformed returns rather than absolute ones. This view forms the basis of the more advanced models used, *e.g.*, for option pricing [40], [41]. Similar traditions are also established in some other fields of science.

Fortunately, characterizing log-normal variation, by 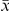 * **^x^**/s*, is no more difficult than using the common description by 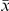 ± SD. Thus, there is no reason why the log-normal should, as has been well expressed by Aitchison & Brown, remain the *Cinderella* of distributions, dominated by its famous “normal” sister [17], and the questions arise, in general: “How normal are additive effects?” and “How normal is the normal distribution?” We believe that the shift in emphasis, away from additive to multiplicative effects and from the normal towards the log- or multiplicative normal distribution, is beneficial and necessary. It will lead to advances in the interpretation of data, and improve our understanding of the concepts behind the empirical phenomena in science and life.

### Analysis

The data used in this study were obtained from the literature. Most references were found by browsing through certain issues of renowned journals and scrutinizing the figures displaying data.- All calculations were done with the statistical programming environment R. For obtaining Fig. 2, a function was written that simulated the power of the t-test on untransformed data for any given sample size *n* and multiplicative standard deviation s*. For given *n>n_0_*, the s* leading to 90% power was then calculated by an ad-hoc method for solving the respective implicit equation.
